# Antenatal breastmilk expression for women with diabetes in pregnancy - a feasibility study

**DOI:** 10.1186/s13006-021-00393-1

**Published:** 2021-07-23

**Authors:** Maren Johnsen, Claus Klingenberg, Meta Brand, Arthur Revhaug, Gunnbjørg Andreassen

**Affiliations:** 1 Department of Obstetrics and Gynecology, Division of Surgery, Oncology and Women’s Health, University Hospital of North Norway, Tromsø, Norway; 2 Paediatric Research Group, Faculty of Health Sciences, UiT-The Arctic University of Norway, Tromsø, Norway; 3 Department of Paediatrics and Adolescence Medicine, University Hospital of North Norway, Tromsø, Norway; 4 Department of Digestive Surgery, Institute of Clinical Medicine, UiT-The Arctic University of Norway, Tromsø, Norway; 5 Division of Surgery, Oncology and Women’s Health, University Hospital North Norway, Tromsø, Norway

**Keywords:** Pregnancy, women’s satisfaction, women’s experience, Breastfeeding, Newborn, Infant, Hypoglycemia, Expressed breast milk, Diabetes, Antenatal period

## Abstract

**Background:**

Mothers with diabetes are less likely to achieve successful breastfeeding. Antenatal breastmilk expression (ABE) may facilitate earlier breastfeeding, but feasibility of introducing ABE and its acceptance among Scandinavian women have previously not been investigated.

**Methods:**

This observational trial was conducted between the 1 January 2019 and the 12 March 2020 in Tromsø, Norway. We aimed to determine the feasibility of ABE in terms of practicality and acceptability among women with medically (metformin or insulin) treated diabetes. Women were invited to participate during antenatal visits from 32 weeks gestation. Participants received instruction and started ABE from gestation week 37 + 0. Participants, and their infants, were followed until 6–8 weeks after birth. We collected data on breastfeeding rates, infant hypoglycemia, transfer to the neonatal unit, and the women’s overall experience and satisfaction with antenatal breastmilk expression.

**Results:**

Twenty-eight of 34 (82%) invited women consented to participate. All started ABE from week 37 + 0, and continued until hospital admission. No women reported any discomfort or side effects. Labor was induced at 38 weeks gestation. Twenty-four women brought harvested colostrum to the maternity ward, which was given to their infants during the first 24 h of life. Breastfeeding rates at discharge were 24/28 (86%) and 21/27 (78%) at 6–8 weeks after delivery. Seven (25%) infants were transferred to the neonatal unit; four because of hypoglycemia. Maternal satisfaction assessed 6–8 weeks after delivery revealed that all participants felt positive about the ABE, but one woman would not recommend it to other pregnant women.

**Conclusions:**

Implementing a structured ABE guideline for women with medically treated diabetes was feasible. The intervention was associated with high level of satisfaction among study participants. No obvious side effects were observed, and breastfeeding rates at discharge and 6–8 weeks after delivery were higher than in comparable studies.

**Trial registration:**

The study was registered at the research study registry at the University Hospital of North Norway (Nr 2018/7181).

## Background

Mothers with diabetes are less likely to achieve successful breastfeeding than mothers without diabetes [1–3]. They have delayed onset of lactation and delivery is often before term due to planned induction. If induction fails there is an increased risk of cesarean section (CS) [4, 5]. Infants of mothers with diabetes are at risk of hypoglycemia due to fetal-neonatal hyperinsulinism. Maternal-child separation after CS or transfer of the infant to a neonatal intensive care unit (NICU) due to hypoglycemia, further decreases the chances of establishing successful breastfeeding [6]. Early provision of infant formula is therefore relatively common in this group of infants, and is generally associated with a shorter time of exclusive breastfeeding [7]. The mother’s own colostrum, is optimal to facilitate the metabolic transition after birth and stabilizes infant glucose concentration more effectively than infant formula [8]. Breastfeeding has numerous benefits including preventing infants from becoming overweight and preventing the development of established diabetes in the mother and the child later in life [9, 10]. In Norway, 89% of all mothers, report that they are breastfeeding (74% exclusively) 8 weeks after birth. Thus, there are clear expectations among pregnant women in Norway that they should breastfeed after birth [11].

Antenatal breastmilk expression (ABE) is the hand expression and collection of colostrum during pregnancy. The benefit of ABE is to have a supply of colostrum for these infants at risk of hypoglycemia whose mothers are at risk of delayed lactogenesis in order to avoid the use of infant formula [1]. It has been studied and implemented as routine care in Australia and New Zealand in order to avoid using infant formula and facilitate earlier breastfeeding [12, 13]. In contrast, albeit being sporadically suggested, ABE is not widely recommended nor studied in Europe, except from in the UK [14]. In our hospital, we experienced that women with diabetes became aware of ABE through the internet or other sources and requested support for this practice. Therefore, we decided to conduct an observational pilot study to assess the feasibility of introducing a clinical guideline for ABE for pregnant women with diabetes. The primary outcome was maternal satisfaction and breastfeeding rates, both at hospital discharge and 6 to 8 weeks after birth. Secondary outcomes were number of infants given harvested colostrum during the first 24 h of life, infant hypoglycemia and NICU admission rates.

## Methods

### Setting and study design

This study was performed in the tertiary care obstetric department at the University Hospital of Northern Norway, Tromsø, Norway. Within the hospital’s catchment area there around 2000 births per year, in three hospital locations. Pregnant women with medically treated diabetes mellitus are advised to give birth only at the tertiary care unit in Tromsø. During the study period, pregnant women with diabetes were routinely admitted to the labor ward at gestational week 38 + 0. If there were no signs of spontaneous labor, induction was initiated with a cervical ripening balloon catheter.

From 1 January 2019, women with diabetes mellitus in need of medication and with a singleton pregnancy were eligible for inclusion in the study. Exclusion criteria were a history of previous preterm birth, antepartum hemorrhage or placenta previa. Women identified as eligible received an invitation to participate during routine antenatal visits from 32 weeks gestation.

Women who consented to participate attended a consultation with one of the project-midwives (MJ, GA, MB) 2–3 weeks before hospital admission for birth induction. During this consultation, the women received practical training in the form of a single session lasting around 1 h. Information leaflets and videos explaining how to perform ABE were provided. The women were given a package with small medicine cups, 2- and 5-ml syringes with stoppers, barcodes and zip bags for storing colostrum. They were informed about good hand hygiene and how to store colostrum safely in their home freezers until hospital admission.

The women were encouraged to stimulate and hand express colostrum three to five times daily for at least 10 min, starting from 37 + 0 week. If they experienced any discomfort, in particular uterine contractions, or had any questions about the intervention, they were asked to contact one of the midwives in the project. The women brought the frozen colostrum to the hospital when they were admitted for induction and/or delivery. The colostrum was stored in a freezer at the postnatal ward, thawed to room temperature in a cup with warm water and given to their infant during the first 24 h after birth.

For infants born to mothers with diabetes the following routine care was applied; early skin-to-skin contact and breastfeeding as the first meal within the first hour after birth, when possible. The first blood sugar was routinely obtained around 2 h after birth, or earlier if hypoglycemia symptoms occurred. Target blood sugar values were > 2.0 mmol/L in the first 24 h and thereafter > 2.6 mmol/L. All infants with blood sugar < 1.4 mmol/l were transferred to the NICU. If blood sugar was between 1.4–1.9 mmol/L infants were first given 5–10 ml expressed milk, pasteurized donor milk or formula from a cup. The blood sugar level was subsequently monitored at least three times during the first 24 h after birth. When stable values > 2.6 mmol/L were reached, monitoring was discontinued and normal postnatal routines with self-regulation continued.

We recorded rates of breastfeeding, both exclusive and partial since birth, at hospital discharge and 6–8 weeks after delivery, when the women were interviewed telephonically. This interview also included two questions, that were rated on a 5-point Likert scale; i) how women had perceived participating in the ABE-project and ii) whether they would recommend ABE to other women. Participants were also given the opportunity to comment openly on the intervention and their experience with antenatal breastmilk expression.

### Data collection, sample size and statistical analyses

Data was collected from the birthplace computer system “Partus” (Clinsoft: Clinical software, Oslo, Norway). No formal sample size calculation was performed for this feasibility study, but we aimed to recruit a convenience sample of 40 women. We had to stop the recruitment on 12 March 2020 when all clinical trials in our hospitals were put on hold due to the COVID-19 outbreak. Categorical data are displayed as number and frequency (%) and continuous data as mean and standard deviation (SD). Data were analyzed using IBM-SPSS (version 26) statistical software.

### Trial registration and ethical approval

The study was registered at the research study registry at the University Hospital of Northern Norway (Nr 2018/7181), and approved by the Regional Ethics Committee (2018/1679 REK Nord).

## Results

During the 14-month study period we invited 34 eligible women and 28 (82%) consented to participate. Clinical data on the mothers and their infants are presented in Table 1. Around two-thirds of the women were nulliparous. The majority had a body mass index above 25 kg/m^2^. Twenty-one participants (75%) had gestational diabetes, treated with metformin or insulin. Mean birthweight of infants in our study was 3349 g, which is between the 25-50th centile for babies born in week 38 in Norway. Most infants had normal Apgar scores. In four out of 28 infants, the first blood sugar was below 1.4 mmol/l (Table 1).

**Table 1:** Clinical data on 28 mothers with medically treated diabetes (metformin or insulin), and their infants

**Mothers**	
Age, years	31.4 (7.1)
Age (range), years	20–45
Parity	
Para 0	18/28 (64)
Para 1+	10/28 (36)
Body mass index (kg/m^2^) ^X^	30.9 (8.8)
Body mass index > 25 kg/m^2^	21/28 (75)
Diabetes mellitus	
Type 1	3 (11)
Type 2	4 (14)
Gestational	21 (75)
Labor	
Labor induction	22/28 (79)
Age induction (gestational week)	38 (0.7)
Vaginal: caesarean delivery	18: 10
**Infants**	
Birthweight, grams	3349 (484)
Apgar score-5 min ≥ 7	25/28 (89)
Blood sugar	
First blood sugar (mmol/L)	2.9 (1.4)
First blood sugar < 1.4 mmol/L	4/28 (14)
Second blood sugar (mmol/L)	3.4 (0.8)
Third blood sugar (mmol/L)	3.5 (0.8)
Admission neonatal unit	7/28 (25)

All values are mean (standard deviation) or number and percentage (%)

^X^ Body mass index at first antenatal consultation

All of the women started ABE from week 37 + 0, and continued until hospital admission. None of the participants reported any discomfort or side effects. Two women contacted the project midwives one time, and requested further advice in regards to the practice of antenatal breastmilk expression. Maternal satisfaction was assessed 6–8 weeks after delivery in 27 of 28 participants; one woman was lost to follow-up. At this time all participants (100%) were positive to the practice of ABE, and 26/27 (96%) would recommend ABE to other pregnant women. In the open-ended question, the vast majority stated that participating in the study and performing ABE was a very positive experience (detailed data not shown). The single woman who did not want to recommend ABE to other mothers perceived ABE as “too laborious”.

**Fig. 1:**
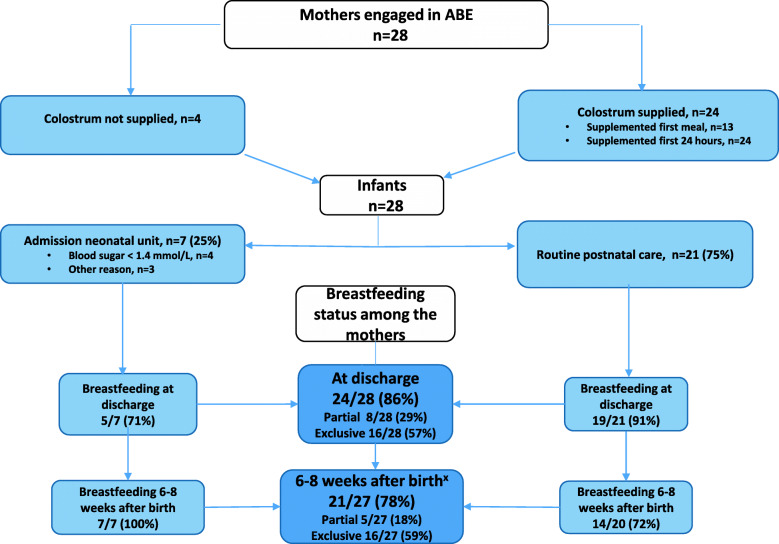
Flow chart – Women with diabetes who started antenatal breast milk expression around 1 week before delivery (*n* = 28) and their infants. ^X^One woman and her infant was lost to follow-up at 6–8 weeks after delivery

Figure 1 shows the flow of the 28 study participants and their infants. Twenty-four women (86%) brought harvested colostrum to the maternity ward. One woman forgot the colostrum at home, and three women stimulated the breasts at home but did not harvest any colostrum. Twenty-four of the 28 infants were breastfed as the first meal after birth. Thirteen infants were also given mean (range) 3 (1–7) ml harvested colostrum as supplement after the first breastfeeding meal. The harvested colostrum was subsequently given to all 24 infants during the first 24 h at different times, when the mother and the midwife felt it was needed to supplement after breastfeeding. Six to eight weeks after delivery 21/27 (78%) mothers were exclusively or partially breastfeeding (Fig. 1). The breastfeeding rate among those admitted to the NICU was 100% at later follow-up.

## Discussion

In this study we have shown that most pregnant women with diabetes who received training in ABE 2 to 3 weeks before admission to the maternity unit were able to produce and harvest colostrum. No adverse effects were reported, in particular no signs of preterm contraction. Most women breastfed upon hospital discharge and also 6–8 weeks after delivery. Participants were positive towards ABE and the intervention, and they would recommend it to other pregnant women with diabetes. Antenatal breastmilk expression thus shows promise of being successful in this population who often face challenges associated with establishing breastfeeding. Moreover, ABE is a simple and well-accepted method to increases maternal skills.

One concern about ABE is that an oxytocin release could potentially lead to early onset of labor. None of the participants in this study reported any contractions related to antenatal breastmilk expression. Other reports have also suggested that there is no harm in advising women to express their own milk before labor and no uterine hyperstimulation was observed [12, 15]. An explanation may be that in a normal pregnancy, the uterine sensitivity to oxytocin and the level of oxytocin receptor level in the myometrium increase up at full-term and are not activated earlier [16]. It is also known that in a normal pregnancy, intercourse or breastfeeding an older sibling is not contraindicated, although oxytocin is also released in these situations [17, 18].

Women with diabetes often feel a higher emotional stress compared to the healthy mothers, and sometimes their concern is exacerbated by healthcare workers [19]. They may also experience guilt and stress, considering the added risk of hypoglycemia to their infant [20]. Maternal empowerment is an important aspect of perinatal care and breastfeeding support [21]. Initiating ABE may empower the woman and subsequently increase her chance for successful breastfeeding and reduce the risk of infant hypoglycemia. We were motivated to do this study after we received requests regarding ABE from many pregnant women. Interviews with the participants indicated a high level of maternal satisfaction with the intervention. In a UK study, women with obesity who were informed about ABE also found it helpful to prepare for breastfeeding [14]. Moreover, the intervention is in line with the “Ten steps to successful breastfeeding”, as defined by the WHO and UNICEF [22], and supports the nursing staff’s aim to empower mothers and enable breastfeeding.

An important goal of this study, was to promote and support breastfeeding after birth among women with diabetes, since they are at increased risk for a delayed lactation. Our results show that 24 of the 28 women breastfed upon discharge and 21/27 (78%) were still breastfeeding 6 to 8 weeks after birth. This is a high rate of breastfeeding among women with diabetes compared to earlier studies, where only around 30–60% of women were breastfeeding at discharge or 6 weeks later [2, 3]. Unfortunately, we do not have Norwegian data on breastfeedng for this specific population. Still, we believe that prenatal education of ABE increased the awareness of delayed lactogenesis among mothers with diabetes in our study, which motivated these women to continue breastfeeding despite initial challenges. Earlier studies also point out that women with diabetes who started ABE were physically and mentally better prepared for breastfeeding than those who did not [23].

In this feasibility study seven (25%) infants were admitted to NICU after birth, but only four due to hypoglycemia. It is worth noting that all second and third blood sugar values obtained from infants in this study were normal during the first 24 h. Our study is small and not powered to assess rates of hypoglycemia or NICU admission. In the large Diabetes and Antenatal Milk Expressing (DAME) multicenter, randomized controlled trial (RCT), the proportion of infants admitted to NICU was lower than in our study, but did not differ between groups assigned to ABE or standard care [12]. We observed that women brought small volumes of colostrum after performing ABE for only around 1 week. DAME study participants started expressing in week 36, 1 week earlier than in our study. In New Zealand they usually advise women to start expressing milk from week 34 [12]. Our results support the notion that it is probably beneficial to start ABE earlier than week 37.

The strengths of this study were the structured approach to ABE. The participants expressed a high degree of satisfaction, and the overall breastfeeding rate was high. The main limitation of this study is that is it not a RCT. The low numbers of participants, exaggerated by early study termination due to the COVID-19 pandemic, precludes strong conclusions regarding infant hypoglycemia and NICU admission rates. Accurate volumes of harvested colostrum were not recorded, but the stimulation of breast milk production before birth may be the most important component of ABE contributing to facilitate breastfeeding shortly after birth. The women were highly satisfied with their inclusion in this ABE study, but this could also be part of the Hawthorne effect, that the study participants feel better because they receive more attention than routine care [24].

## Conclusions

We found that implementing a structured ABE guideline for women with medically treated diabetes was feasible and associated with high level of satisfaction among study participants. No obvious side effects were observed, breastfeeding rates at discharge and 6–8 weeks after birth were higher than in comparable studies. Antenatal breastmilk expression may lead to increased maternal empowerment and increased breastfeeding rates among women with diabetes or other women with known challenges in establishing breastfeeding. Well-designed, and adequately powered RCTs with appropriate outcomes, including user perspective data, are needed to define the role of ABE in future perinatal care.

## Data Availability

The dataset from the current study is available from the corresponding author on reasonable request.

## Abbreviations

ABE: Antenatal breastmilk expression
NICU: Neonatal Intensive Care Unit
RCT: Randomized Controlled Trial
